# Rosacea in childhood and adolescence: A review

**DOI:** 10.1111/ddg.15693

**Published:** 2025-03-22

**Authors:** Sören Korsing, Karola Stieler, Uwe Pleyer, Ulrike Blume‐Peytavi, Annika Vogt

**Affiliations:** ^1^ Department of Dermatology Venereology and Allergology Charité – Universitätsmedizin Berlin corporate member of Freie Universität Berlin and Humboldt‐Universität zu Berlin Berlin Germany; ^2^ Department of Ophthalmology Charité – Universitätsmedizin Berlin corporate member of Freie Universität Berlin and Humboldt‐Universität zu Berlin Berlin Germany; ^3^ Berlin Institute of Health at Charité – Universitätsmedizin Berlin Berlin Germany

**Keywords:** Demodex, IFAG, Pediatric Dermatology, Rosacea

## Abstract

Despite presenting with similar symptoms, triggers, and progression patterns as adults, rosacea in children and adolescents is frequently overlooked as a primary differential diagnosis. However, initial manifestations of classic clinical types can be observed from infancy onwards. The frequent association of childhood rosacea with ophthalmologic manifestations is of particular importance in addition to the knowledge of special subtypes such as idiopathic aseptic facial granuloma.

Therapeutic options of pediatric rosacea partly overlap with those of common differential diagnoses such as acne vulgaris or perioral dermatitis. However, both the disease itself and its manifestation in this age group entail challenges in the selection of active ingredients and the development of therapeutic concepts. In addition, advice on triggering and aggravation factors is required in order to provide optimal long‐term support for those affected.

## EPIDEMIOLOGY AND PREDISPOSING FACTORS

Rosacea is a chronic inflammatory disease that often occurs in adults between the ages of 30 and 50 and primarily affects the central facial skin. Rosacea is less frequently considered in children and adolescents, but is probably underdiagnosed or misdiagnosed.^1^ This may be due to the lower awareness of the disease in this age group, morphological similarities with the much more common acne vulgaris or clinical differences compared to adulthood (e.g., idiopathic facial aseptic granuloma or periorificial variant).

Precise data on incidence and prevalence do not exist. The first manifestation usually occurs between the ages of 4 and 8. It often takes several months to years before a diagnosis is made. The predisposing factors seem to correspond to those of adult patients: light photobiological skin type I–II according to Fitzpatrick, particularly in the papulopustular variant, as well as the use of topical or inhaled steroids, particularly in the periorificial variant.^2^, ^3^ Occurrence in children and adolescents with darker skin types is possible.^4^, ^5^ However, it is not yet clear whether the incidence is really lower in darker skin types or whether there is a diagnostic gap.^6^ The data on gender preference is inconsistent, but mostly shows an even distribution.^7^ Some studies show a higher incidence in female patients, particularly in severe ophthalmorosacea.^2^

## PATHOGENESIS

According to current knowledge, the pathogenesis of rosacea in children and adolescents does not differ from that in adults and is based on predisposing genetic factors and external trigger factors. Dysregulation of the innate and acquired immune system, vasomotor instability, and interaction with microorganisms are pathogenetically relevant.^8^ Regardless of age, ultraviolet radiation, heat, spicy or hot foods and drinks, topical steroids and psychological stress are seen as trigger factors for the manifestation of rosacea.^9^

As in adults, a familial predisposition to rosacea may be present in pediatric patients.^10^ Single candidate genes and two single nucleotide polymorphisms associated with rosacea have been identified.^11^ The presence of a gain‐of‐function mutation in the *STAT1* gene results in an immunodeficiency with associated overgrowth of Demodex mites. The leading dermatological symptom of this immunodeficiency can be the early appearance of rosacea‐like lesions.^12^, ^13^

Cathelicidins play a central role at the molecular level. These antimicrobial peptides are released by mast cells, inducing proinflammatory mediators such as interleukin (IL)‐8 and promoting neovascularization via stimulation of vascular endothelial growth factor (VEGF). The increased presence of cathelicidins in the skin of rosacea patients is due to the increased production of precursor molecules and increased expression of matrix metalloproteases such as kallikrein.^14^, ^15^ Cathelicidins are induced via Toll‐like receptor (TLR)2, which is overexpressed in rosacea.^16^ Microorganisms such as *Staphylococcus epidermidis* and Demodex mites are involved in the initiation of an immune response in rosacea via the activation of TLR2.^17^ Immunocompetent children with healthy skin are generally not infested with Demodex mites. Colonization increases during adolescence.^18^ Successful therapy approaches based on a reduction of Demodex colonization in children with rosacea suggest a pathophysiological connection in this age group as well.^19^ However, the detection of Demodex mites is not obligatory in rosacea patients.

The adaptive immune system is also involved in the pathogenesis of rosacea in the form of an immune response dominated by type 1 T helper cells. This results in the upregulation of other proinflammatory cytokines such as tumor necrosis factor (TNF)‐α and IL‐17.^20^

Finally, neurovascular dysregulation plays a relevant role. In individuals with a corresponding predisposition, the typical trigger factors of rosacea lead to increased activation of transient receptor potential vanilloid 4 (TRPV4) ion channels.^21^

## DIAGNOSIS AND CLINICAL PRESENTATION

**FIGURE 1:**
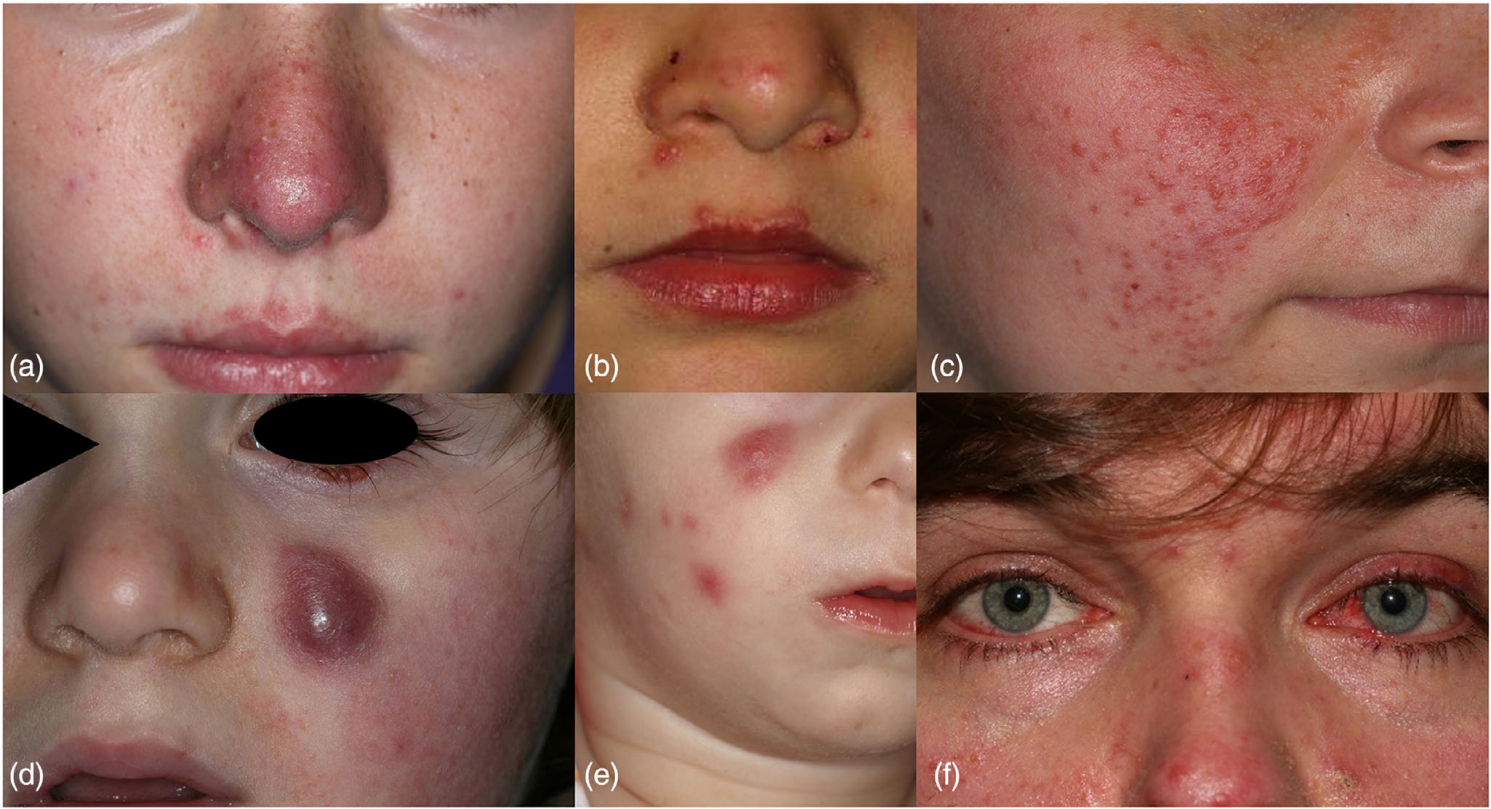
Clinical phenotypes of rosacea in childhood and adolescence: (a) combined erythematous rosacea (ER) and papulopustular rosacea (PPR), (b) periorificial rosacea (POR), (c) granulomatous rosacea (GR), idiopathic facial aseptic granuloma (IFAG) with (d) solitary lesion and cheek erythema, as well as (e) multiple lesions, (f) ophthalmorosacea (OR).

The diagnosis of rosacea is based on clinical findings, which in pediatric patients can closely resemble those in adults. While epidemiological data indicate that, across all age groups, 57% of rosacea cases are erythematotelangiectatic, 43% are papulopustular, 7% are phymatous, and 11% are ocular, precise data on pediatric cases are lacking.^6^ From the perspective of published treatment series in children, however, it becomes apparent that periorificial manifestations, ocular manifestations as well as severe types of idiopathic facial aseptic granuloma present particular clinical challenges in these age groups compared to the common forms of erythematotelangiectatic and papulopustular rosacea (Table 1, Figure 1).^22^ The phymatous form has not yet been described in the young age group.^23^

**TABLE 1:** Subtypes and characteristics of rosacea in childhood and adolescence.

**Subtype**	**Characteristics**
Erythematous or telangiectatic rosacea (ER)	Facial flushes and/or persistent erythema
Papulopustular rosacea (PPR)	Papules and pustules on convex areas of the face (cheeks, forehead, chin)
Periorificial variant (POR)^*^	Periorificial localization of papulopustules, usually perioral with involvement of the philtrum
Granulomatous variant (GR)	Firm papules and nodules, granulomatous infiltrate Idiopathic facial aseptic granuloma
Idiopathic facial aseptic granuloma (IFAG)^*^	Solitary or multiple erythematous to livid nodules
Ophthalmorosacea (OR)^*^	Blepharitis, conjunctivitis, chalazia, hordeola or corneal affections (keratitis, neovascularization, ulcers)

* While precise data on the proportions of the subtypes in children and adolescents are lacking, own experience and the presentation in case series and treatment reports underline that the highlighted subtypes pose reoccurring and sometimes therapeutically challenging entities in these age groups.

To date, no internationally approved diagnostic criteria for rosacea in children and adolescents have been defined.^24^ However, due to the overlap with adulthood, we refer to the current S2k guideline for the diagnosis.^9^ Chamaillard et al. propose the following criteria in pediatric patients, whereby two criteria must be present for diagnosis:

‐ Flushing or persistent facial erythema
‐ Facial telangiectasias that cannot be explained by other causes
‐ Papulopustular lesions, absence of comedones
‐ Localization of the lesions on convex areas of the face
‐ Ocular involvement, including blepharitis, recurrent chalazia, conjunctivitis, and keratitis^7^

In addition, subjective symptoms such as pruritus, burning or stinging and a feeling of dryness may occur.^9^ Biopsies or other further diagnostics are generally not required and are used to rule out other differential diagnoses. There are few case reports on the histopathological characteristics of pediatric rosacea patients. However, these do not appear to differ from those of adults.^25^ Special tape removal methods using a cyanoacrylate adhesive can provide evidence of increased Demodex colonization in some cases, but should be reserved for selected cases in view of the sensitivity of the patient's skin.^26^ As the appearance varies according to the clinical type, a different spectrum of possible differential diagnoses results.

### Erythematous or telangiectatic rosacea (ER)

The flushes last longer than a few minutes or persist, and therefore differ from physiological erythema. They particularly affect the cheek region. Flushes can be triggered by heat or UV light. Macroscopically visible telangiectasias may occur.^1^, ^3^, ^7^ The clinical presentation of ER may precede the development of papulopustular lesions.^10^

### Papulopustular rosacea (PPR)

Papulopustular rosacea is the most frequently described variant in childhood and adolescence. Overlaps with ER and periorificial rosacea are possible (Figure 1a). Subjective symptoms such as pruritus, dysesthesia, or skin burning are rarely described.^1^, ^7^ Acne vulgaris may be present at the same time.

Induction or exacerbation by steroids is possible (steroid rosacea). Rosacea fulminans in adults is defined as a rare, acute maximum variant of papulopustular rosacea, characterized by nodules, abscesses and fistulae and is described particularly in pregnant women. There are a few case reports of rosacea fulminans in adolescents and one case report of a 3‐year‐old female patient.^27^, ^28^

### Periorificial rosacea (POR)

The papulopustular lesions usually show a perioral distribution with involvement of the philtrum (Figure 1b). Other variants are localized perinasally or periocularly. It is not possible to differentiate it from classic perioral dermatitis, which is induced by excessive skin care and topical steroids. Some authors postulate that perioral dermatitis in children and adolescents should always be classified as rosacea. This is supported by the fact that clinical and histopathological differentiation is not possible and both conditions respond to the same treatment principles.^29^, ^30^

### Granulomatous rosacea (GR)

The variant is described extremely rarely and presents clinically as follicle‐associated, brown‐red nodules (Figure 1c). Under glass spatula pressure, an apple jelly‐like infiltrate may appear. The histopathology shows granulomas.^31^

### Idiopathic facial aseptic granuloma (IFAG)

Idiopathic facial aseptic granuloma is considered a variant of GR and manifests in infancy as solitary (Figure 1d) or rarely multiple (Figure 1e), erythematous nodules that are typically located infraorbitally or on the cheeks. Peripheral signs of inflammation are absent. There is usually no subjective discomfort. Children often present after surgical incision or biopsy.^32^, ^33^

Sonography can be helpful for diagnosis and follow‐up.^34^ The histology shows lymphohistiocytic infiltrates, folliculitis or granulomas in varying degrees. Spontaneous regression without scarring or recurrence usually occurs after about 1 year, so that a wait‐and‐see approach can be considered, although varying responses to topical or systemic antibiotics or isotretinoin in severe cases have been reported.^35^ Affected children may show further clinical signs of rosacea according to Chamaillard et al.^7^, ^35^ This phenotype does not occur in adults. In some cases, distinguishing it from inflamed cysts or tumorous processes, such as pilomatricomas, may be challenging. Therefore, knowledge of this entity is helpful to avoid unnecessary surgical interventions.

### Ophthalmorosacea (OR)

**FIGURE 2:**
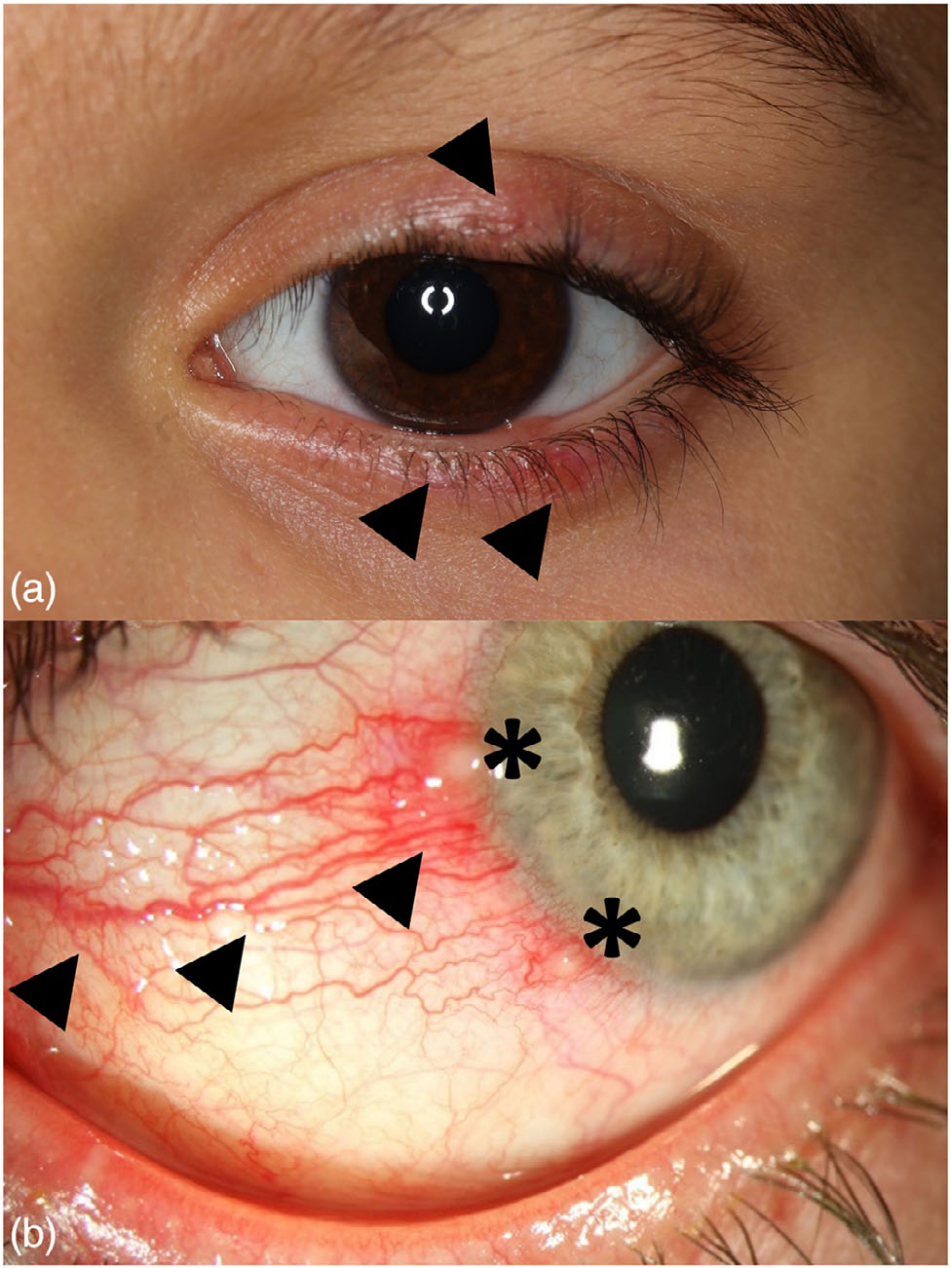
Findings in ophthalmorosacea (OR): (a) blepharitis with lid erythema and papulovesicles of the lower lid (arrow). (b) Severe blepharoconjunctivitis with pronounced neovascularization (arrow) and ulcers (asterisk).

Ocular lesions occur in up to 60% of children and adolescents, and is more common than in adults.^2^, ^36^, ^37^ Ophthalmorosacea is often bilaterally localized (Figures 1, 2).^38^ Female children and adolescents appear to have more frequent and severe symptoms and report dryness and foreign body sensation, burning and tearing, photosensitivity, redness of the conjunctiva and eyelids, or swelling of the orbital and periorbital region.^2^ In about half of young patients, the ocular symptoms and findings precede the cutaneous signs.^7^, ^39^ In addition, these can occur separately, which makes the diagnosis more difficult. If there is a combined oculocutaneous rosacea, there is often a papulopustular phenotype.^40^

The predominant initial clinical change is meibomian gland dysfunction with inflammation spreading to the ocular surface (conjunctivitis, keratitis). Slit‐lamp examination of the eyelid margins reveals telangiectasia, dilated meibomian glands with secretion retention and “collarettes”. Meibomian gland dysfunction results in disorders of the lipid composition of the tear film with increased tear evaporation, reduced tear film break‐up time and evaporative dry eye. Often, recurring inflammations and redness at the lid margin can provide an important additional diagnostic clue to differentiate from acne vulgaris.^41^

### Morbihan's disease

**FIGURE 3:**
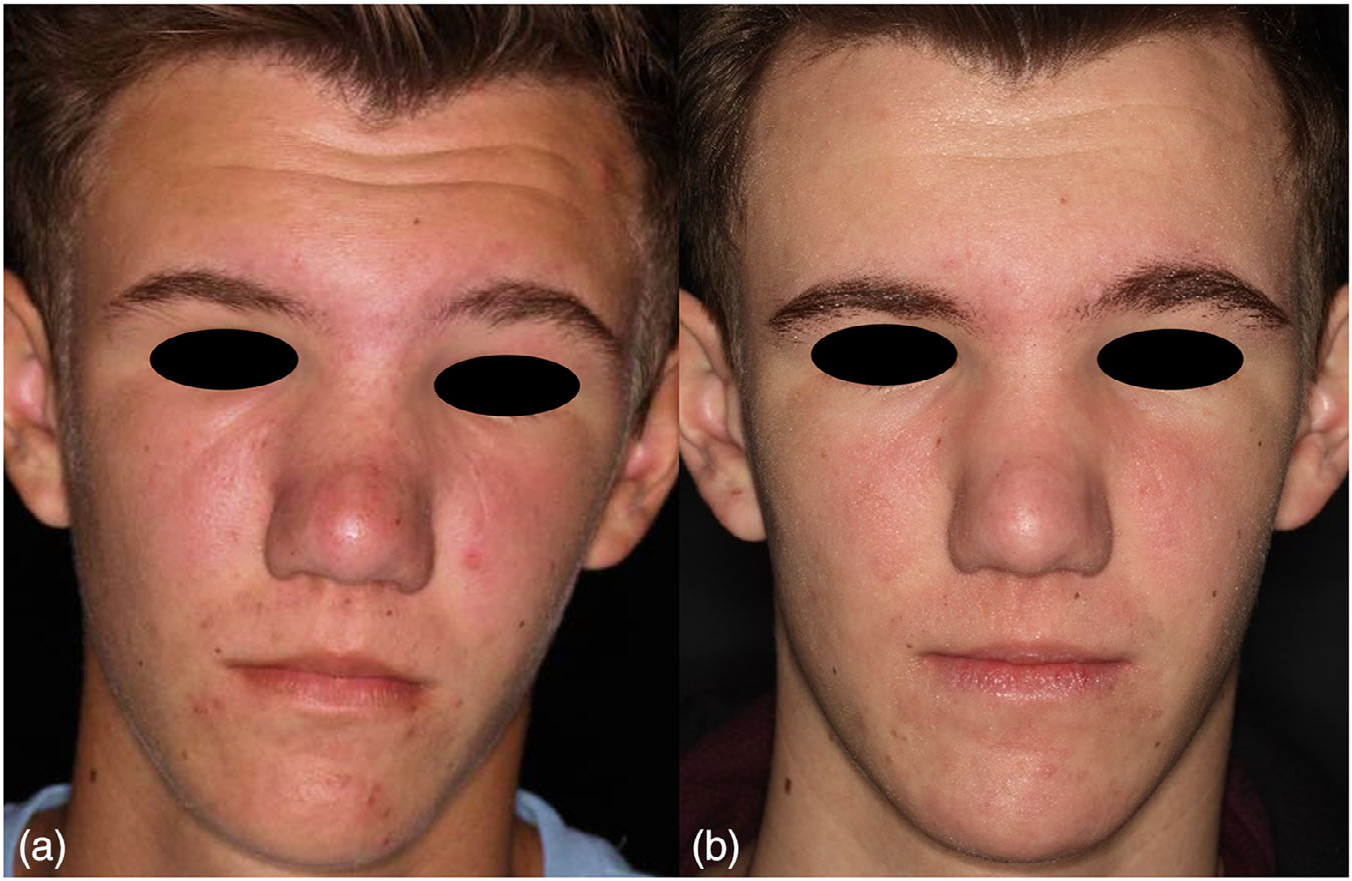
Morbihan disease: (a) 15‐year‐old adolescent with papulopustular lesions of the cheeks and perioral region, firm, cushion‐like swelling of the cheeks, nose, and glabellar region with significant subjective tightness. (b) Marked improvement in papulopustules, erythema, and swelling reduction after three months of systemic therapy with isotretinoin and manual lymphatic drainage.

This is a special variant of rosacea that presents with persistent, cushion‐like swelling of convex and periocular areas of the face with or without further inflammatory efflorescence. Men are more frequently affected.^6^, ^42^ To our knowledge, there are no case reports of Morbihan's disease or facial lymphedema in connection with rosacea in pediatric patients. However, in our clinical experience, although rare, moderate lymphedema can also be observed in this patient group (Figure 3).

## DIFFERENTIAL DIAGNOSES

Rosacea in children and adolescents is uncommon, necessitating the exclusion of a wide range of differential diagnoses based on clinical manifestations.

Since IFAG typically presents as single lesions in infants, the main differential diagnoses include pilomatrixomas, juvenile xanthogranulomas, and Spitz nevus. Other differential diagnoses include epidermal cysts, chalazia, infectious dermatoses (e.g., mycobacteriosis and cutaneous leishmaniasis), and foreign body granulomas.^3^

Gianotti‐Crosti syndrome and benign cephalic histiocytosis should be considered in infants and toddlers when disseminated facial lesions are present. In cases of persistent facial erythema, consideration should be given to butterfly erythema in systemic lupus erythematosus or ulerythema ophryogenes.

During adolescence, acne vulgaris is a key differential diagnosis for papulopustular lesions, characterized by the presence of open or closed comedones, but it is distinguished from rosacea by the absence of facial flushing, persistent erythema, and telangiectasia.

Other potential differentials across all age groups include irritant‐toxic and allergic contact dermatitis, seborrheic dermatitis, demodicosis (particularly in immunocompromised patients), sarcoidosis, and granulosis rubra nasi.

In ocular rosacea, viral or bacterial infections, irritant‐toxic or allergic causes or atopic dermatitis should be considered.

## THERAPY

The treatment of rosacea in children and adolescents is largely based on the therapeutic strategies for adults, while taking into account the specific needs of young patients. Both in children and adults, rosacea treatment focuses on alleviating symptoms, reducing inflammation, and improving quality of life and is modified depending on the clinical phenotype and severity. Essentially, the recommendations of the current S2k guideline, which also refers to manifestations in young patients, can be reproduced.^9^ However, a particular challenge is the fact that all therapeutics are used off‐label, as they are either only approved for adults or not primarily indicated for rosacea. Additionally, age limits must be considered in the selection of therapeutics, such as the use of tetracyclines.

Basic measures include the identification and avoidance of trigger factors such as heat, UV light, or irritating external agents including steroids. Furthermore, the use of mild cleansers and soothing external agents is recommended. UV protection measures should be applied during the day.^9^

Topical therapy is employed for mild to moderate severity, while systemic therapies in combination with topical measures are used for severe cases. There is sparse data available for the therapeutics used in pediatric patients, relying on individual case reports, case series, and retrospective data analysis.^22^, ^43^ Tables 2 and 3 provide an overview of available therapeutics.

**TABLE 2:** Topical preparations for the treatment of cutaneous rosacea in children and adolescents (selection).

Ready‐to‐use preparation	Possible dosage for rosacea in children and adolescents	Approval for rosacea in adults	Approval in children and adolescents	Reference
Azelaic acid 15% (gel)	2 times daily for 4 – 12 weeks	Yes	Yes (acne vulgaris)	*44*
Brimonidine 0.3% (gel)	Once daily as needed	Yes	No	*2*
Erythromycin 2% (solution)	2 times daily for 4 – 12 weeks	No	Yes (acne vulgaris)	*33*
Ivermectin 1% (cream)	Once daily for 12 weeks	Yes	No	*2* *^,^* *45*
Pimecrolimus 1% (cream)	Once daily until improvement	No	Yes (atopic dermatitis)	*46*
Tacrolimus 0.03% or 0.1% (ointment)	Once daily until improvement	No	Yes (atopic dermatitis)	*46*
Metronidazole 0.75% (gel, lotion, cream)	2 times daily for 12 weeks	Yes	No	*2* *^,^* *3* *^,^* *30* *^,^* *48*

* The availability and approval regulations pertain to Germany.

**TABLE 3:** Systemic therapies of cutaneous rosacea in children and adolescents (selection).

Pharmaceutical	Possible dosage in childhood and adolescence	Approval for rosacea (in adults)	Approval in childhood and adolescence	Reference
Azithromycin	10 mg/kg BW/day (on 3 consecutive days of the week)	No	Yes (acute infections)	^48^
Clarithromycin	15 mg/kg BW/day	No	Yes (acute infections)	^32^, ^33^
Erythromycin	30–50 mg/kg BW/day	No	Yes (acute infections)	^2^, ^7^, ^25^, ^40^, ^50^
Doxycycline	2.2–4.4 mg/kg BW/d from 8–11 years of age. 40–100 mg/d from the age of 12	Yes	Yes, from the age of 9 (acute infections)	^2^, ^7^
Isotretinoin	0.1–0.3 mg/kg/d	No	Yes, from the age of 12 (acne vulgaris)	^28^, ^51^, ^52^
Ivermectin	200–250 µg/g as a single dose	No	Yes, from 15 kg (scabies)	^19^, ^45^

* The availability and approval regulations pertain to Germany.

### Topical therapy

The topical use of metronidazole in pediatric patients, particularly in PPR and POR, has demonstrated efficacy, especially when combined with systemic antibiotics.^2^, ^3^ However, topical metronidazole is not approved for use in children. Azelaic acid can also be effectively used in pediatric patients.^44^ The transient skin burning sensation at the beginning of therapy should be considered, especially in pediatric use. Initially reducing the frequency of application with gradual increase every other day may be helpful. In a small cohort of pediatric patients with PPR and POD, topical ivermectin showed good efficacy with good tolerability.^45^ The advantage of once‐daily application is noted. Topical calcineurin inhibitors are another treatment option, well‐tolerated, and may be considered, particularly in steroid‐induced rosacea.^46^ Other topical preparations include erythromycin, clindamycin, permethrin, and benzoyl peroxide.^47^

### Systemic therapy

Severe and extensive forms of rosacea in childhood and adolescence may require systemic therapies. These are typically combined with topical measures over a period of 8 to 12 weeks. Macrolides, tetracyclines, metronidazole, isotretinoin, and ivermectin are mentioned in the literature.^2^, ^45^ Macrolides can be safely used before the age of 9. Azithromycin at a dosage of 10 mg/kg body weight for approximately 6 weeks was applied in a case series of 222 children with POD, showing good therapeutic response and tolerability.^48^ Intermittent intake on three consecutive weekdays appears advantageous here. Alternatives include erythromycin and clarithromycin.^2^, ^33^, ^49^ Therapy with macrolide antibiotics may also be considered for IFAG.^33^, ^50^ Doxycycline can be used in children with rosacea from the age of 9 onwards. In younger patients, this is contraindicated due to the risk of enamel or bone defects.^2^

There are only a few case reports on treatment attempts with isotretinoin in children with severe disease manifestation. Low‐dose therapy for approximately 6 months resulted in a good therapeutic response.^28^, ^51^ Furthermore, isotretinoin therapy led to rapid remission of IFAG after 2 months.^52^ Single doses of ivermectin in children and adolescents with PPR were well tolerated, with the exception of temporary skin scaling at the beginning of therapy, and showed good efficacy.^45^

### Treatment of ophthalmorosacea

For the treatment of OR, a combined long‐term treatment regimen is often necessary. Basic measures for OR include eyelid hygiene and artificial tears. Topically, antibiotics (azithromycin, moxifloxacin, gentamicin), steroids (hydrocortisone), and cyclosporine are mentioned in the literature. Starosta et al. describe in a small case series the intermittent use of azithromycin eye drops 1.5% in combination with eyelid hygiene.^53^

Based on the idea that *Demodex folliculorum* plays a role in the development of blepharitis, local and systemic measures are recommended (tea tree oil‐soaked wipes, oral ivermectin, sometimes combined with oral metronidazole).^9^

Systemic therapy is carried out using macrolides (azithromycin, erythromycin, roxithromycin), tetracyclines (doxycycline, minocycline), or metronidazole for at least 3 to 6 months.^49^, ^54^

A single dose of ivermectin also resulted in complete remission in a 12‐year‐old patient with severe PPR with ocular involvement.^19^ Interdisciplinary diagnosis and therapy involving dermatologists and ophthalmologists should be pursued.

### Device‐based treatment options

There is limited evidence for the use of device‐based treatment options therapeutic measures such as lasers or intense pulsed light (IPL) in pediatric patients with rosacea. However, the use of these treatments in adolescents is conceivable, in the same way as in adults.^9^

## CLINICAL COURSE AND PROGNOSIS

The majority of pediatric patients who received temporary systemic therapy remained without recurrence in the subsequent years.^2^, ^33^ It remains unclear whether affected children and adolescents are at risk of developing rosacea again in adulthood.^10^ Further studies to assess long‐term outcomes are desirable.

## CONCLUSIONS

- Diagnosis of rosacea in childhood and adolescence is based on clinical findings, which often resemble the presentation of the disease in adults.
- Special forms in childhood and adolescence include periorificial rosacea or idiopathic facial aseptic granuloma (IFAG).
- Awareness of IFAG is important to avoid unnecessary diagnostic and surgical interventions.
- Children and adolescents are more frequently affected by ocular involvement, which must be recognized, appropriately treated, and monitored. Interdisciplinary therapy involving ophthalmologists is recommended.
- All therapeutic measures are conducted off‐label.
- Topical therapy options include azelaic acid, brimonidine, calcineurin inhibitors, erythromycin, ivermectin or metronidazole.
- In cases of severe disease manifestation, systemic therapy with macrolide antibiotics (erythromycin, azithromycin), metronidazole, or from the age of 9 onwards, doxycycline may be required. Ivermectin and isotretinoin may be considered.

## CONFLICT OF INTEREST STATEMENT

A.V. is advisor and lecturer for Amryt, Bayer Healthcare, Galderma Laboratorium GmbH, Pfizer, Sanofi Regeneron. U.B.‐P. is an advisor, lecturer or performed clinical studies for Abbvie, Amryt, Bayer Healthcare, Boots Healthcare, Cantabria Labs, Cassiopeia, CeraVe, Concert Pharmaceuticals, Dermocosmétique Vichy, Galderma Laboratorium GmbH, Eli Lilly, LEO‐Pharma, Novartis, Mayne Pharma, Pfizer, Pierre Fabre, Sanofi Regeneron. U.P. is consultant for Abbvie, Alcon, Allergan, Alimera, Bausch und Lomb, Bayer, Novartis, Santen, Thea. S.K. and K.S. state that there are no conflicts of interest.
